# Sample-to-Answer Immuno-Magnetic Assay Using Thermally Responsive Alkane Partitions

**DOI:** 10.3390/bios12111030

**Published:** 2022-11-17

**Authors:** Micaela L. Everitt, David J. Boegner, Ian M. White

**Affiliations:** Fischell Department of Bioengineering, University of Maryland, College Park, MD 20742, USA

**Keywords:** point-of-care, magnetofluidics, COVID-19

## Abstract

To combat pandemics, there is a need for rapid point-of-care diagnostics to identify infected patients and to track the spread of the disease. While recent progress has been made in response to COVID-19, there continues to be a need for point-of-care diagnostics capable of detecting biomarkers—such as antibodies—in whole blood. We have recently reported the development of thermally responsive alkane partitions (TRAPs) for the automation of point-of-care immuno-magnetic assays. Here, we demonstrate the use of TRAPs to enable sample-to-answer detection of antibodies against the SARS-CoV-2 virus in whole blood samples. We report a limit of detection of 84 pg/mL, well below the clinically relevant threshold. We anticipate that the TRAP-enabled sample-to-answer immunoassay can be used to track the progression of future pandemics, leading to a more informed and robust clinical and societal response.

## 1. Introduction

More than any other event in history, the COVID-19 pandemic raised awareness about the debilitating lack of diagnostic capabilities in all regions of the world. Early in the pandemic, a lack of testing led to uncontrolled community spread and uncertain levels of disease pervasiveness due to untraced cases. As technology evolved to meet the demands of the pandemic, both viral and serological testing played important roles in tracking those infected. Serological testing is particularly helpful for tracking cases where patients were not tested during the acute infection stage, which can be especially useful in a pre-vaccination stage of a pandemic. Moreover, during the initial vaccine rollout, serological testing can help to validate vaccination technologies. Because access to these serological diagnostic tools can have dramatic effects on the spread of disease, increasing access for the current pandemic and for future pandemics is necessary. To accomplish this, serological diagnostics should be point-of-care such that they can be performed in near-patient settings, such as a physician’s office or walk-in clinic, such that no technical expertise or bulky equipment is required. Specifically, the diagnostic should be sample-to-answer, meaning that it should require no user intervention or precise sample manipulation steps [1].

Traditionally, detecting antibody-based biomarkers requires an enzyme-linked immunosorbent assay (ELISA), which can take hours to perform. Furthermore, ELISA-based diagnostics are confined to central laboratories due to the need to perform precise technical steps. Efforts to transition ELISAs to the point-of-care have most notably resulted in lateral flow immunoassays (LFIAs). In LFIAs, a sample is wicked along an ensemble of membranes, where an immuno-sandwich forms at a specific location when the biomarker is present; a tag on the labeling antibody (often gold nanoparticles) results in a visible line in the readout location. LFIAs work well for non-complex samples, such as a nasal swab. However, to be used with whole blood, the sample must first either be centrifuged to recover the plasma from the blood sample, or the whole blood must be diluted by several orders of magnitude. These additional precise steps preclude the assays from being used at the point of care. Thus, while LFIAs for SARS-CoV-2 antibodies have been published [2,3,4] and some even have Emergency Authorization Use (EAU) from the Food and Drug Administration [5], they require precise whole blood preparation steps, preventing them from point-of-care use. Although progress has been made to integrate whole blood sample preparation in LFIAs via serum separation pads, underlying issues remain, including erythrocyte clogging, high filtration pressure, and slow flow rate [6,7]. These issues are caused by small membrane pore size; however, increasing the pore size causes poor separation efficiency and decreased serum purity.

The field of microfluidics has offered numerous solutions aimed at point-of-care diagnostics [8,9]. Some microfluidic devices have demonstrated sample-to-answer capabilities but are either prohibitively expensive due to complex manufacturing or they introduce manual steps to swap out external tubing [10,11]. Alternatively, the field of magnetofluidics aims to automate sample preparation steps for reagent manipulation with the simplicity of an external magnet. In magnetofluidic systems, magnetic micro- or nanoparticles within or on a cartridge are manipulated by an external magnet to transport bound molecules through the steps of an assay [12,13,14,15,16,17,18], thus eliminating manual reagent handling steps.

For example, in Shin et al., the authors use magnetic beads manipulated by an external magnet to capture viral RNA and transport it from one well to another, following the steps of nucleic acid recovery and PCR amplification [19]. Magnetic beads can also be used to capture and transport protein biomarkers through an immunoassay. Mani et al. have engineered a magnetofluidic immunoassay for the detection of antibodies in plasma [20]. In this design, reagents incorporated into a cartridge are separated using immiscible oil. Through immunocapture, the magnetic beads capture the biomarker from the sample and pull the biomarker through the steps of the immunoassay under the control of an external magnet. This innovative design eliminates the manual reagent transfer steps of typical immunoassays. However, the oil-based partitioning is likely not stable under mechanical agitation, such as vibrations from shipping and typical handling in the field.

Recently, we reported a novel method for automated sample and reagent manipulation using thermally actuated pseudo-valves constructed of higher order alkanes [21,22,23,24]. Higher order alkanes have nine or more carbon atoms and have a high viscosity when liquified. They are solid at room temperature and liquid at slightly elevated temperatures. When deposited between reagents, these thermally responsive alkane partitions (TRAPs) serve to partition prepackaged reagents in an assay cartridge and then enable sample manipulation when liquified (via heat). Unlike oil, the TRAPs are stable under severe mechanical agitation [25]. We have demonstrated that we can use TRAPs in two distinct modes for sample-to-answer assay development: (1) as a removable partition for hands-free reagent mixing following melting and (2) as a continual partition that separates assay regions while enabling magnetic beads to be pulled through following melting, enabling hands-free immuno-magnetic assays [25]. This TRAP behavior can be controlled through the specific geometry of the millimeter-scale channels in which the TRAPs are confined. For instance, TRAPs breach upon melting when using thinner TRAPs and/or broader channels, and thus reagents flanking the TRAP mix following the thermal actuation. Conversely, the TRAPs remain stationary upon melting when using thicker TRAPs and/or narrower channels, and thus reagents flanking the TRAP remain separated following the thermal actuation.

**Figure 1:**
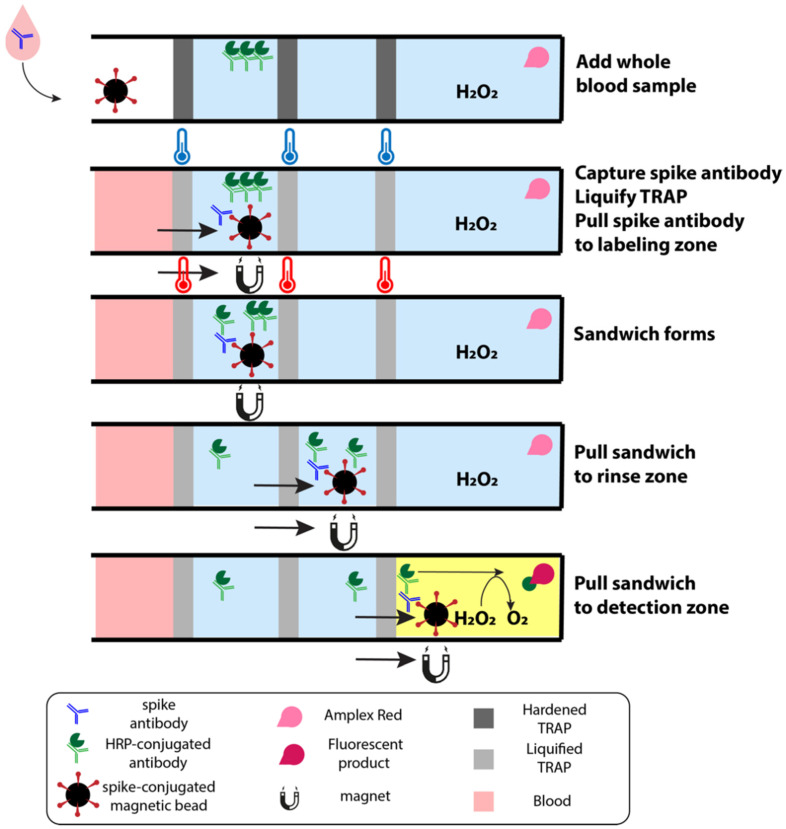
Schematic of sample-to-answer immunoassay from whole blood. The first zone houses magnetic beads functionalized with SARS-CoV-2 spike proteins that capture antibodies against the spike protein in the whole blood sample. As the cartridge is heated, the TRAPs liquify, allowing an external magnet to pull the beads through the first TRAP to the second zone. HRP-conjugated secondary antibodies in this second zone label any antibodies captured by the beads. These complexes are pulled through rinses to shed any labels that are caught in the hydration layer of the bead slug before reaching the detection zone. The detection zone contains hydrogen peroxide and Amplex Red, which fluoresces in the presence of HRP.

In the present work, we use this second geometric configuration to construct a sample-to-answer immuno-magnetic assay to quantify antibodies against the SARS-CoV-2 spike protein in whole blood samples. In our assay, a capillary blood sample is added to a cartridge containing several reagent zones, separated by TRAPs (Figure 1). These zones include a sample zone in which antibodies against the SARS-CoV-2 spike protein are captured onto magnetic beads, a labeling zone where the captured antibodies are labeled with an enzyme-tagged secondary antibody, rinse zones to remove any excess labels, and a detection zone to fluorescently detect the enzyme-tagged label. After melting the TRAPs that separate the zones, magnetic beads are pulled by an external magnet from one zone to the next, through the permeable TRAPs, thus transporting the antibodies against the SARS-CoV-2 spike protein through the immunoassay and into the detection zone where they can be quantified.

Using the TRAP-enabled system, we show distinguishable fluorescence measurements with a limit of detection of 84 pg/mL and a dynamic range that extends up to 1 μg/mL. In the literature, approximately 1 ng/mL [26,27,28] is used as the clinical threshold cutoff to be considered positive for antibodies against the spike protein of SARS-CoV-2. However, based on many variables that can impact immunity against viruses (number of vaccinations, number of infections, number of exposures, time since vaccination, time since infection, time since exposure, overall health, etc.), ranges for anti-spike antibodies from 1 ng/mL–200 μg/mL in blood have been reported [29]. Thus, our sample-to-answer assay enables clinically relevant detection of antibodies for SARS-CoV-2 spike protein directly from whole blood samples. Moreover, this system can be easily modified to detect additional antibodies in blood at the point-of-care, which may be important in the next pandemic. 

## 2. Materials and Methods

### 2.1. Magnetic Bead Preparation

The 1 μm streptavidin magnetic beads (Pierce) are prepared by gathering 100 μL 10 mg/mL beads to the side of the tube using an external magnet, aspirating out their buffer, and rinsing them with 200 μL of wash buffer, comprised of 25 mM Tris and 150 mM NaCl (both from Sigma-Aldrich, St. Louis, MO, USA). The beads are again gathered to the side of the tube using an external magnet, and the wash buffer is removed. Furthermore, 50 μL 200 μg/mL SARS-CoV-2 biotinylated spike receptor binding domain (RBD) protein (ProSci) is added to the 1 mg washed beads and left to incubate for 1 h at room temperature. Following this incubation, the beads are washed three times by magnetically gathering them to the side of the tube, aspirating out the supernatant, and washing them with 200 μL of wash buffer. On the final rinse, 25 μL of 0.1 M phosphate buffer is added to the mass of beads, resulting in 40 mg/mL magnetic beads coated in SARS-CoV-2 spike RBD protein.

### 2.2. Cartridge Construction

The cartridges with channels (3 × 3 × 47 mm^3^) are 3D printed with Prusament UV sensitive resin from Prusa Research. Once cured, a coverslip (Fisher Scientific, Waltham, MA, USA) is glued to the open face of the cartridge, covering the channel, and left to dry overnight at room temperature. To ensure a hydrophobic surface, we incubate 423 μL glass water repellent (Rain-X) in the cartridge for 30 min at room temperature. Following incubation, excess glass water repellent is removed, and the cartridges are washed three times with water.

### 2.3. TRAP Assay Layer Construction

Eicosane (melt temperature = 42 °C), a higher order alkane, is used to form the TRAPs to separate each reagent layer. First, to construct the assay, the cartridges are held vertically and filled with 50 μL solution containing 5 μM Amplex Red (Biotium, Fremont, CA, USA) and 1 mM hydrogen peroxide (Fisher Scientific). To prepare eicosane (Alfa Aesar, Haverhill, MA, USA), it is first melted by placing it in a glass vial on a hot plate at 120 °C. Furthermore, 30 μL of melted eicosane quickly hardens as it is deposited by a micropipette atop the Amplex Red layer. Then, 60 μL 0.1 M phosphate buffer is added, then another 30 μL of melted eicosane, then 60 μL 0.1 M phosphate buffer, then 30 μL of melted eicosane, then 50 μL 100 ng/mL horseradish peroxidase (HRP)-conjugated anti-rabbit IgG antibodies (ThermoFisher), then 30 μL of melted eicosane. Finally, in the top zone, 2.5 μL 40 mg/mL magnetic beads coated in SARS-CoV-2 spike RBD protein is added.

### 2.4. Blood Sample Preparation

Whole blood is withdrawn from an exposed vessel at the elbow pocket of swine forelimbs within 15 min after the animal is euthanized. The blood is collected into an EDTA-coated collection tube, which is then repeatedly inverted to mix the blood and EDTA. The tube is then stored at 4 °C. Immediately preceding the experiment, the blood is spiked with varying concentrations (0–1000 ng/mL) of rabbit SARS-CoV-2 spike RBD protein antibodies (ThermoFisher). In addition, 50 μL spiked blood samples are added to the top zone of the TRAP assay. All work performed with animals was completed with approval from University of Maryland, College Park Institutional Animal Care and Use Committee (R-AUG-20–47).

### 2.5. Portable Fluorescence Measurements

**Figure 2:**
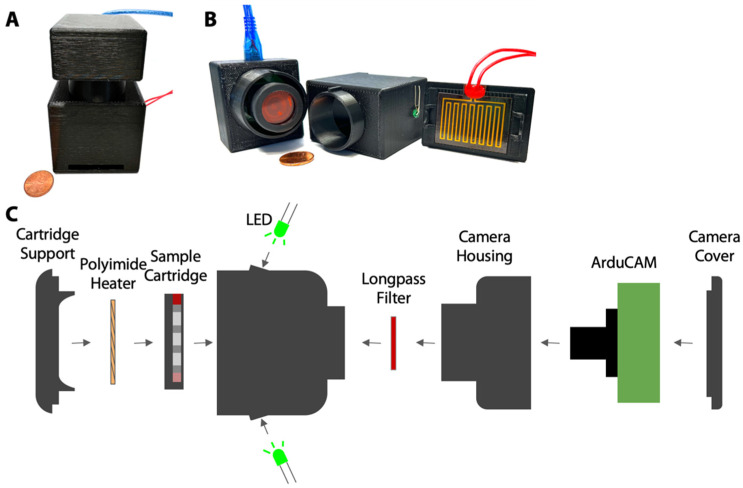
Schematic of portable fluorescence reader with built in heater. (**A**) The ArduCAM, LEDs, filter, and heater are housed in a 3D printed handheld device. (**B**) The device opens to separate the heater from the ArduCAM to insert an assay cartridge. (**C**) The ArduCAM is housed behind a longpass filter to detect fluorescence produced from a sample, which is excited by LEDs built into the device above the sample cartridge. The cartridge is placed on top of a polyimide heater to liquify the TRAPs.

Fluorescence measurements are taken by placing a cartridge into a portable fluorescence reader designed by our lab, depicted in Figure 2. The fluorescence reader is comprised of 3D printed parts that secure in place an ArduCAM MT9M001 Camera with an ArduCAM USB2 Camera Shield (ArduCAM), a 570 nm longpass filter (Thorlabs, Newton, NJ, USA) on the lens of the camera, two 525 nm LEDs to excite the sample, and a polyimide heater. The sample cartridge positions the sample in a precise location relative to the camera.

### 2.6. Image Processing

Image processing is performed using a MATLAB script that automatically crops an image to a 39 × 39-pixel window. This window only contains the region of interest along a cartridge channel. Fluorescence values reported in this paper are generated by calculating the mean of the set of all grayscale pixel values within the pixel window.

### 2.7. Portable Heating

The heat required to melt eicosane in each device is supplied via a polyimide heating pad that is adhered to the 3D printed support that holds the sample cartridge in place (Figure 2). Upon placing a sample cartridge into its support, 6V DC is supplied across the polyimide heater. Within two minutes of switching the power on, the temperature of the heater reaches eicosane’s melting point (Figure S1, Electronic Supporting Information).

### 2.8. Sample-to-Answer Immunoassay

After the blood sample is added to the top zone of the assay and left to incubate for 30 min, the cartridge is placed on the portable heater to melt the eicosane layers. Once melted, an external neodymium magnet (25.4 × 6.35 × 6.35 mm^3^) gathers and pulls the beads across the first layer of eicosane into the zone containing HRP-conjugated antibodies. The heater is turned off and the beads are left to incubate for 30 min. After incubating, the heater is turned back on to melt the eicosane layers. Then, an external magnet regathers and pulls the beads across the second layer of eicosane, into one of the zones containing phosphate buffer. Here, the beads are released temporarily in the rinse zone to help promote rinsing. The beads are subsequently magnetically gathered and pulled across the third layer of eicosane, into the other zone containing phosphate buffer, and released temporarily. Finally, the beads are pulled across the fourth layer of eicosane into the zone containing Amplex Red and hydrogen peroxide. The heater is turned off, and the fluorescence is measured by the portable device 10 min after the beads reach the final zone.

### 2.9. Manual Bead-Based Immunoassay

We performed a bead-based ELISA with manual wash steps by adding 2.5 μL 40 mg/mL magnetic beads coated in SARS-CoV-2 spike RBD protein to 50 μL whole blood samples spiked with SARS-CoV-2 spike RBD protein antibodies (0–1000 ng/mL). The beads and antibodies are left to incubate at room temperature for 30 min. The beads are gathered to the side of the tube using an external magnet, the supernatant is aspirated out, and the beads are rinsed with 200 μL wash buffer three times. On the final rinse, the beads are resuspended in 50 μL 100 ng/mL HRP-conjugated anti-rabbit IgG antibodies and left to incubate at room temperature for 30 min. The beads are again gathered to the side of the tube using an external magnet, the supernatant is aspirated out, and the beads are rinsed with 200 μL of wash buffer three times. On the final rinse, the beads are resuspended for 5 min in 5 μL elution buffer, which is comprised of 0.1 M glycine (Sigma-Aldrich) at pH 2. Then, 5 μL of supernatant is collected and added to a 50 μL solution containing 5 μM Amplex Red and 1 mM hydrogen peroxide. Fluorescence resulting from manual bead washing is measured using a Synergy LX plate reader from BioTek (530 nm excitation, 590 nm emission, Winooski, VT, USA). After the eluted sample is added to the Amplex Red/hydrogen peroxide solution, the liquid is moved to a 96-well plate. A fluorescence measurement is taken after 10 min.

### 2.10. Capillary Tube Blood Collection

We have implemented a cap for the TRAP cartridge with a blood collection capillary (RAM Scientific, Nashville, TN, USA). To collect blood, the fingertip is cleaned and pricked with a lancet. Then, the capillary touches the blood droplet. The blood is quickly wicked into the capillary tube. Once the capillary is full, the blood is loaded into the cartridge by vertically tapping the device. The capillary cap is then removed and replaced with a closed cap. A video of this process can be found in the Electronic Supplement.

## 3. Results and Discussion

**Figure 3:**
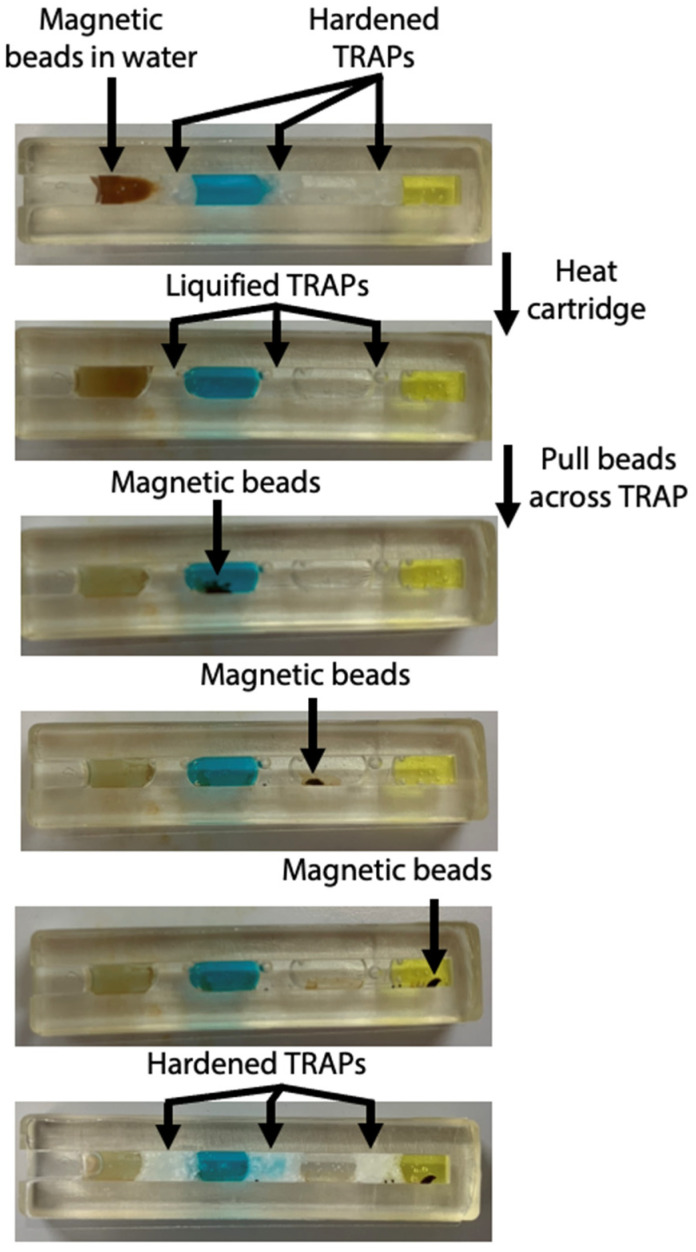
Photos of magnetic beads pulled through TRAPs in cartridge. The cartridge is heated to melt the TRAPs. Once liquified, the magnetic beads are pulled through each of the permeable barriers. The cartridge is removed from heat and the TRAPs harden.

We have recently shown that we can use TRAPs as stationary partitions that continually separate reagents but are permeable to magnetic beads, thus enabling magnetic microbeads to transport bound molecules across the partition [25]. Here, we have specifically demonstrated use of TRAPs in our sample-to-answer magnetofluidic assay to quantify antibodies against the spike protein of SARS-CoV-2. Our assay contains five separate assay sections (1 capturing zone, 1 labeling zone, 2 rinse zones, 1 detection zone), thus our final design requires four stationary TRAPs that are permeable to magnetic beads (Figure 3). The first zone houses magnetic beads functionalized with SARS-CoV-2 spike proteins to capture anti-spike antibodies in the blood sample. Upon melting the TRAPs, the antibodies captured on magnetic beads are pulled through the permeable TRAPs by an external magnet, thus transporting the antibodies from whole blood into another aqueous layer containing HRP-conjugated secondary antibodies, which bind to the captured SARS-CoV-2 spike protein antibodies. This complex is pulled through another permeable TRAP and into the phosphate buffer. This rinse zone greatly dilutes any unbound secondary labeling antibodies pulled through the TRAP in the hydration layer around the bead slug. This automation allows us to minimize the number of manual steps, which are required in the gold standard equivalent (ELISA). Finally, the bead complex is pulled into a detection zone containing Amplex Red and hydrogen peroxide. HRP (tagged onto the labeling antibody) converts Amplex Red into fluorescent resorufin, thus producing a fluorescent signal that is directly proportional to the concentration of anti-spike antibodies. In this work, the total assay time is 70 min; however, this can be greatly decreased in a commercial device by optimizing the incubation times.

We have chosen to use a channel with a 3 × 3 mm^2^ cross-section within the cartridge, as we previously established that this geometry is narrow enough for the TRAPs to remain stationary as they are melted. As evidenced by Figure 3, the width of the cartridge allows the magnetic beads to pass through the TRAPs without causing them to breach. The layers of the assay remain separated during melting of the TRAPs, magnetic bead transit across the TRAPs, and rehardening of the TRAPs.

We aim to use our sample-to-answer assay in a near-patient setting, which requires the entire device to be portable and inexpensive. We have developed a handheld instrument to both heat the TRAPs and to read the resulting fluorescence. The optical components of this instrument are based on our previous work [24], in which we demonstrated a detection limit comparable to a benchtop plate reader. The current iteration of this instrument includes a built-in heater (Figure 2) to automatically melt the TRAPs after the cartridge is inserted. The heater is comprised of a polyimide heating pad adhered to the 3D printed support that holds the cartridge, such that, without moving the cartridge, the TRAPs can be melted, and the camera can take fluorescence images. When the cartridge is placed into its support, 6V DC is supplied across the heater for 2 min, after which the heater stabilizes at 62 °C (Figure S1, Electronic Supporting Information). Characterization experiments demonstrated that this external temperature quickly melts the eicosane (Tm = 42 °C) TRAPs inside the channel. Previous experiments showed that this temperature did not disrupt antibody binding in the channel [25].

**Figure 4:**
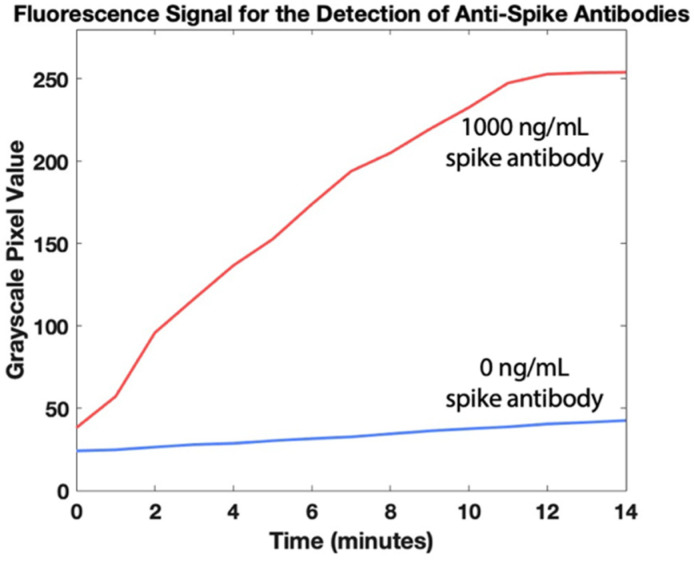
Fluorescence quantification of antibodies against SARS-CoV-2 spike protein in whole blood over 14 min. The heater is turned off at t = 0 min, right after the magnetic beads reach the detection zone.

After optimization of the cartridge geometry and assay conditions, we ran the full assay to analyze blood samples spiked with a range of concentrations of antibodies against the SARS-CoV-2 spike protein. Following a 30-min incubation once the whole blood sample is added to allow beads to capture antibodies against the SARS-CoV-2 spike protein, the cartridge is placed in the portable fluorescence reader atop the portable heater. The bead slug is pulled through the immunoassay and into the detection zone as shown in Figure 3. The heater turns off and the fluorescence is imaged by the camera 10 min after the beads reach the detection zone. The real-time fluorescence data in Figure 4 demonstrates that signal saturation is achieved after about ten minutes.

**Figure 5:**
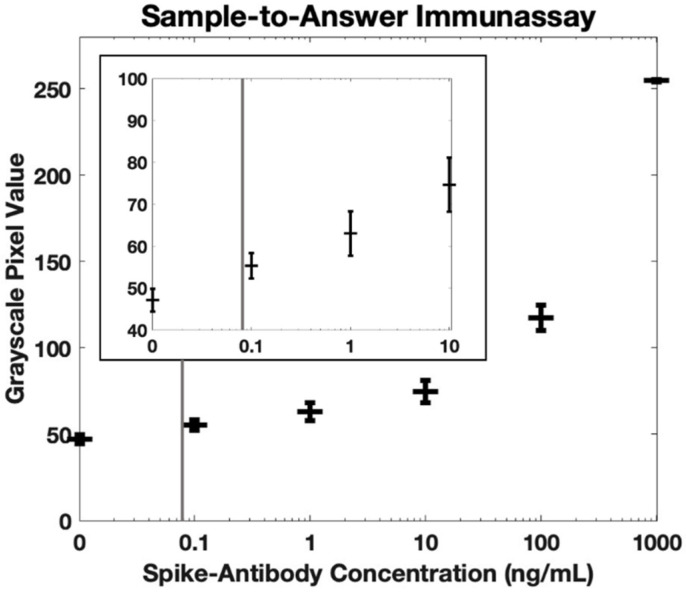
Fluorescence quantification of antibodies against SARS-CoV-2 spike protein in whole blood using our sample-to-answer immunoassay and portable fluorescence reader (*n* = 3). The calculated detection limit is 84 pg/mL using the IUPAC definition (indicated by gray vertical line). Error bars are +/− 1 standard deviation. The insert shows the same dataset but on a smaller scale.

Figure 5 shows the measured fluorescence intensity for antibody concentrations in whole blood between 0 ng/mL and 1000 ng/mL. Using the International Union for Pure and Applied Chemistry (IUPAC) definition of the limit of detection (the concentration that generates a signal with a mean that is separated from the mean of the blank by three standard deviations of the blank), the calculated limit of detection for our sample-to-answer assay is 84 pg/mL. Similarly, using the International Organization for Standardization (ISO) definition for the limit of detection (5% error on the blank and 5% error on a positive sample with the lowest concentration that is identifiable as positive [30]), the calculated limit of detection is 102 pg/mL. All values for detection limit are significantly lower than the reported range of anti-spike antibodies in whole blood [26,27,28,29], demonstrating that this system is clinically relevant.

**Figure 6:**
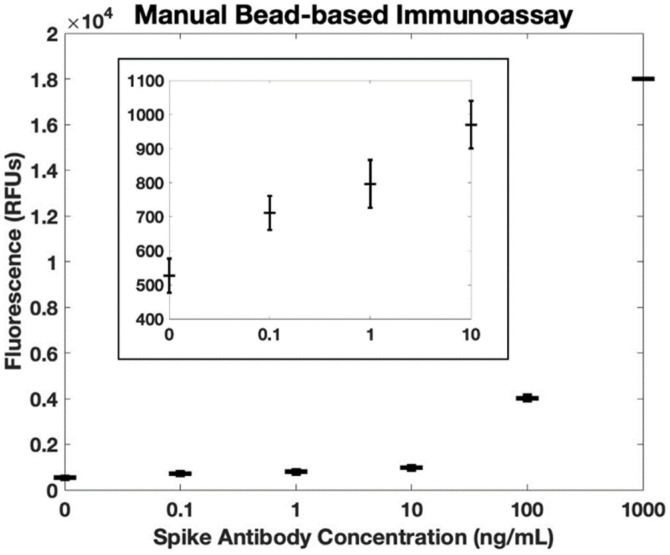
Fluorescence quantification of antibodies against SARS-CoV-2 spike protein in whole blood using a bead-based immunoassay with manual rinse steps and a benchtop plate reader (*n* = 3). The calculated detection limit is 68 pg/mL using the IUPAC definition. Error bars are +/− 1 standard deviation. The insert shows the same dataset, but on a smaller scale.

For comparison with gold standard methods, we performed a manual bead-based immunoassay (manual pipetting steps for reagent manipulation) and used a benchtop plate reader for fluorescence quantification. The whole blood samples were spiked with antibodies against the SARS-CoV-2 spike protein. These samples were incubated with magnetic beads functionalized with SARS-CoV-2 spike proteins. After incubation, the beads were manually washed before incubating with HRP-functionalized secondary antibodies. Following incubation, again the beads were manually washed. Because the beads could not be present when measuring fluorescence in the benchtop plate reader, on the final wash step, the beads were resuspended in elution buffer. Finally, the supernatant was collected and added to a plate containing Amplex Red and hydrogen peroxide. The resulting fluorescence was measured in a benchtop plate reader (Figure 6). Note that the measured fluorescence intensity for 100 pg/mL is more than three standard deviations above the background fluorescence level, confirming that this concentration is within the detectable range. Using the IUPAC definition, the calculated limit of detection of this manual bead-based assay is 68 pg/mL, comparable to our sample-to-answer assay (84 pg/mL). Likewise, using the ISO definition, the calculated limit of detection of the manual bead-based assay is 80 pg/mL, similar to the performance of our sample-to-answer assay (102 pg/mL). Both methods resulted in comparable limits of detection, suggesting that our sample-to-answer assay does not sacrifice sensitivity as it takes on key elements of point-of-care diagnostics.

**Figure 7:**
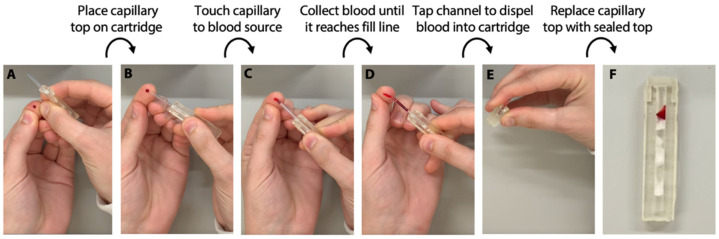
Photos of demonstration of capillary-based cap for automated blood collection. (**A**) First, the capillary cap is placed on the cartridge; then, (**B**) the capillary is placed in contact with the blood sample (e.g., droplet on a fingertip) and (**C**) blood is quickly (i.e., 60 s) wicked into the capillary until (**D**) a precise volume is reached. (**E**) The cartridge is tapped vertically to dispel blood into the cartridge, and (**F**) the capillary cap is replaced with the sealed cap. A video of a whole blood sample being loaded into the cartridge can be viewed in a video included in the Electronic Supporting Information.

The previous experiments demonstrate that this immunoassay is completely automated without sacrificing performance. However, to be truly point-of-care, assays need to be sample-to-answer, implying that untampered samples such as whole blood must be collected and loaded into the cartridge without any precise manual sample transfers. Thus, point-of-care tests cannot rely on venous blood draws performed by phlebotomists and should instead enable the patient to draw their own sample or enable easy collection at a collection site via nurse, lab technician, or physician’s assistant. We have designed our device to pull a precise volume of whole blood directly from a finger stick into the cartridge via a capillary tube. The capillary tube is built into the cap (Figure 7A), which when contacted with the blood sample (Figure 7B), quickly wicks blood (Figure 7C) until a precise volume is reached (Figure 7D). The cartridge is then tapped to empty the blood into the channel (Figure 7E), and the capillary tube cap is exchanged with a sealed cap after the sample is loaded (Figure 7F). This entire process can be completed in 165 s (see the video in the Electronic Supporting Information). The integration of this sample collection step is crucial for sample-to-answer diagnostics as there are no precise manual sample transfer or preparation steps required of the user.

## 4. Conclusions

In this work, we have developed a sample-to-answer assay for the detection of antibodies against the SARS-CoV-2 spike protein in whole blood. We report a detection limit that is below the clinical threshold cutoff to be considered positive for anti-spike antibodies. Fluorescence measurements were taken using a portable reader containing a built-in heater to integrate sample preparation steps into the overall system. Finally, we have also integrated the blood collection step using a built-in capillary tube.

Further work will continue to improve our system. Movement of the external magnet will be motorized for full automation. In addition, the portable fluorescence imager can be further miniaturized by replacing the ArduCam (which is useful for prototyping) with a simple photodetector. We also hope to further optimize the assay by reducing the incubation time required in each zone. Additional future work will broaden the applications of the TRAP-enabled magnetofluidic assay. For instance, other antibody biomarkers can be detected by using a different antigen on the magnetic beads. As an example, in this work, we detected the antibody to the spike protein, which indicates exposure to either the virus or vaccine; in contrast, for an assay to indicate exposure to the virus only, one could use the nucleocapsid antigen. More generally, any biomarker molecule—including protein and DNA sequences—can be detected with this point-of-care technology by utilizing the appropriate capture molecule. Finally, this technology can be deployed in the next pandemic or for other urgent point-of-care blood assays to improve accessibility to testing.

## Data Availability

The data presented in this study are available on request from the corresponding author.

## Supplementary Materials

The following supporting information can be downloaded at: https://www.mdpi.com/article/10.3390/bios12111030/s1, Figure S1: Validation of portable heater; Video S1: Demonstration of blood collection and loading.
