# Supplementary material for: Sample-to-Answer Immuno-Magnetic Assay Using Thermally Responsive Alkane Partitions
Source: Biosensors (Basel). 2022 Nov 17;12(11):1030. doi: 10.3390/bios12111030 (PMC9688217; doi:10.3390/bios12111030)
Supplement: Supplementary file 1 [file biosensors-12-01030-s001.zip › Electronic Supplement.pdf]

Sample-to-Answer Immuno-Magnetic Assay Using Thermally Responsive Alkane Partitions

Micaela L. Everitt [1], David J. Boegner [1] and Ian M. White [1,*]

[1] Fischell Department of Bioengineering, University of Maryland, College Park 20742, United States

* Correspondence: ianwhite@umd.edu

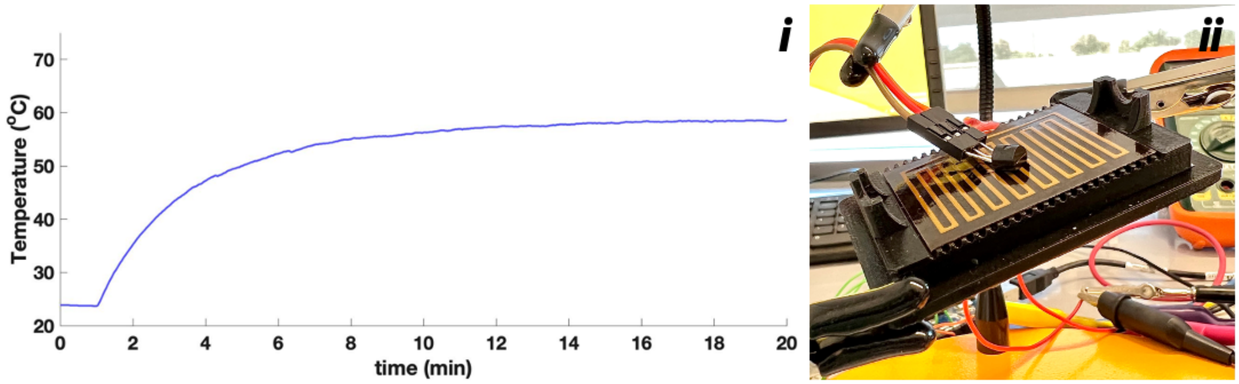

Figure S1: Validation of portable heater. (i) The temperature of the heater is tracked via thermocouple beginning as the heater is turned on. The heater reaches it steady state (62 °C) within 6 minutes. (ii) The thermocouple is placed right up against the heater, precisely where the cartridge would be placed.

Video S1: Demonstration of blood collection and loading. After cleaning the fingertip, a lancet is used to prick the skin. Blood is wicked up the capillary tube cap by placing the capillary in contact with the blood sample. The cartridge is tapped vertically to dispel blood into the cartridge and the capillary cap is replaced with a sealed cap. This entire process can be completed in 165 seconds.
